# Probabilistic Approach for Virtual Screening Based on Multiple Pharmacophores

**DOI:** 10.3390/molecules25020385

**Published:** 2020-01-17

**Authors:** Timur I. Madzhidov, Assima Rakhimbekova, Alina Kutlushuna, Pavel Polishchuk

**Affiliations:** 1 A.M. Butlerov Institute of Chemistry, Kazan Federal University, 420008 Kazan, Russia; timur.madzhidov@kpfu.ru (T.I.M.); asima.astana@outlook.com (A.R.); alina.kutlushina@upol.cz (A.K.); 2 Institute of Molecular and Translational Medicine, Faculty of Medicine and Dentistry, Palacky University and University Hospital in Olomouc, 77900 Olomouc, Czech Republic

**Keywords:** pharmacophores, machine learning, virtual screening, ligand-based virtual screening

## Abstract

Pharmacophore modeling is usually considered as a special type of virtual screening without probabilistic nature. Correspondence of at least one conformation of a molecule to pharmacophore is considered as evidence of its bioactivity. We show that pharmacophores can be treated as one-class machine learning models, and the probability the reflecting model’s confidence can be assigned to a pharmacophore on the basis of their precision of active compounds identification on a calibration set. Two schemes (Max and Mean) of probability calculation for consensus prediction based on individual pharmacophore models were proposed. Both approaches to some extent correspond to commonly used consensus approaches like the common hit approach or the one based on a logical OR operation uniting hit lists of individual models. Unlike some known approaches, the proposed ones can rank compounds retrieved by multiple models. These approaches were benchmarked on multiple ChEMBL datasets used for ligand-based pharmacophore modeling and externally validated on corresponding DUD-E datasets. The influence of complexity of pharmacophores and their performance on a calibration set on results of virtual screening was analyzed. It was shown that Max and Mean approaches have superior early enrichment to the commonly used approaches. Thus, a well-performing, easy-to-implement, and probabilistic alternative to existing approaches for pharmacophore-based virtual screening was proposed.

## 1. Introduction

Pharmacophore modeling is a widely used approach for the discovery of new biologically active compounds. According to the IUPAC definition, pharmacophore is an ensemble of steric and electronic features that is necessary to ensure the optimal supramolecular interactions with a specific biological target and to trigger (or block) its biological response. [1] Once such pharmacophore is found, the task is to find a compound that has the same arrangement of interaction centers, called pharmacophore features, in at least one of the low-energy conformations. This process being done in silico is called pharmacophore-based virtual screening. There are multiple examples of successful applications of pharmacophore models to find hit compounds [2,3]. Previously, a single pharmacophore model was commonly used for virtual screening [4,5]. This model could be derived from a structure of a ligand–protein complex or could be generated from a set of known active compounds. For instance, seven known antibacterial compounds that inhibit bacterial RNA polymerase and have different binding modes were flexibly aligned to find a pharmacophore matching a common binding mode. The final model consisted of four core features (two aromatic, one H-bond donor/acceptor/aromatic, and one anion), one accessory feature (hydrophobic), and two aromatic projections. It was used for screening of an in-house library of 2000 compounds. A total of 64 hits were identified and 11 of them were experimentally confirmed as RNA polymerase inhibitors [6]. In the case in which multiple models of the same target are used for virtual screening, consensus hits—compounds that matched multiple models—could be a preferable way to select hits. It is more likely that a compound would be active if it matches multiple pharmacophores [7]. Kurczab and Bojarski [8] derived a set of possible pharmacophore features from multiple structure-based pharmacophore models of 5-HT7 ligands and enumerated three-, four-, and five-feature models. They found that consensus of these models, when compound is considered active if it corresponds to at least one pharmacophore model, outperforms any single model. This is a widely used consensus screening approach we will refer to hereinafter as OR-consensus. Usually, this consensus scheme works well until there is a poor model in a set of pharmacophore models that retrieves a lot of inactive compounds that may substantially decrease the overall screening performance. Therefore, models should be validated on a dataset of actives and inactives before using them for virtual screening. Only pharmacophores with reasonably high performance should be taken for OR-consensus. Within the described OR-consensus scheme, all models are treated as equal, which does not allow compound ranking. Meanwhile, ranking of hits may improve the performance of virtual screening as it would be possible to select a smaller number of compounds with a higher probability to find actives. Recently, the common hits approach (CHA), which ranked compounds based on the percentage of matched pharmacophore models, was suggested for virtual screening based on pharmacophores retrieved from molecular dynamic (MD) simulations of protein–ligand complexes [9]. Alternatively, the conformer coverage approach was suggested to rank compounds based on the percentage of compound conformers matched MD pharmacophores [10].

We proposed a new approach to virtual screening, which treats multiple ligand-based pharmacophore models according to their individual performances. This approach allows ranking of virtual screening hits and making more precise selection of them.

## 2. Results and Discussion

### 2.1. Pharmacophores and Probabilities: Proposed Approach

Usually, pharmacophore modeling is considered as a special type of virtual screening without a probabilistic nature. Correspondence of at least one conformation of a molecule to pharmacophore is considered as evidence of its bioactivity. However, this is not exactly right; simple pharmacophores having few features usually does not guarantee bioactivity, while correspondence of a molecule to some complex pharmacophores means that molecule will possess the activity with high confidence.

A pharmacophore model from a machine learning (ML) point of view is a typical case of a one-class classification model, which tries to identify objects of a specific class among all objects by learning from a training set containing only the objects of that class [11,12]. Indeed, pharmacophores are designed to extract active compounds and they are generated usually on a subset of active molecules; however, inactive can be taken into consideration upon model generation as refinement of pharmacophores, but not to generate pharmacophores of the inactive class (space of pharmacophores for inactives is considered infinite, and thus is ignored). In such a way, pharmacophore modeling is a chemistry-specific one-class classification method based on abstraction of 3D structure of molecules as a set of features with a given spatial orientation that can be recognized by a target biomolecule.

Similarly to regular classification methods, one can assess the probability that an object belongs to a particular class Y (usually actives class, and so hereafter, Y means actives) if it has a particular pharmacophore $x_{i}$ based on accuracy prediction of a calibration set containing active and inactive molecules:

(1) $$P\left(Y|x_{i}\right)=\frac{N_{Y\cap x_{i}}}{N_{x_{i}}}=Precision\left(x_{i},\;dataset\right),$$

where $N_{Y\cap x_{i}}$ is the number of active compounds among those that were retrieved by the pharmacophore $x_{i}$ and $N_{x_{i}}$ is the total number of retrieved compounds. $P\left(Y|x_{i}\right)$ is nothing else but a precision of the pharmacophore model $x_{i}$ estimated on a calibration set. This probability can be interpreted in an opposite way; that is, as the confidence that a molecule possessing pharmacophore $x_{i}$ is active. This provides an explanation for the intuitively clear concept that pharmacophores with greatest precision should be used for virtual screening.

In the case of multiple pharmacophore models being used for screening, it is important to assess P(Y|X), the probability of a compound to be active based on matching of several pharmacophore models, where X is a set of pharmacophores with $x_{i}$ corresponding to a given molecule. The estimation of P(Y|X) should favor matching of highly accurate models, $P\left(Y|x_{i}\right)\to 1$, and should be insensitive to poor models, $P\left(Y|x_{i}\right)\to 0$. Therefore, for example, geometric and harmonic mean of $P\left(Y|x_{i}\right)$ for a set of pharmacophores, or multiplication of $P\left(Y|x_{i}\right)$ values, can be excluded from consideration owing to high sensitivity to poor performing models.

In our opinion, the following are two the most suitable hypotheses to estimate P(Y|X) based on the performances of individual pharmacophore models $P\left(Y|x_{i}\right)$:

- Max scheme. In this case, P(Y|X) is simply the maximal value of $P\left(Y|x_{i}\right)$:
  (2) $$P\left(Y|X\right)=\max P(Y|x_{i}).$$
  It will be reduced to the OR-consensus strategy (selection without ranking) if $P\left(Y|x_{i}\right)$ is set to 1 for all models. However, using performances of individual models estimated on a calibration set, we can associate athe ctivity of compounds with a probability according to Equation (2).
- Mean scheme. The value of P(Y|X) is an arithmetic mean of $P\left(Y|x_{i}\right)$ over all pharmacophores matching a compound:
  (3) $$P\left(Y|X\right)=\frac{\sum_{i}^{S}P(Y|x_{i})}{S}.$$

This approach is reduced to the common hit approach (CHA) [9] if $P\left(Y|x_{i}\right)$ is set to 1 for all models, and S will be the total number of pharmacophores in the set.

In such a way, having a set of pharmacophores, one can use them all to construct a one-class classification model that can rank new compounds according to probability to retrieve active compounds estimated on a dataset of known compounds. Therefore, the proposed approach requires a set of known active and inactive compounds, which would be used as a calibration set to determine performance (namely precision) of individual pharmacophore models. The advantage of the Max and Mean schemes (Equations (2) and (3)) over the regularly used OR-consensus and CHA approach is that it results in a greater number of distinct values, and thus it can better discriminate selected compounds and improve their ranking.

Unlike approaches used before, we propose the scheme that applies pharmacophores not only as classification models with two outcomes (active/inactive), but probabilistic models that can rank the compound of interest according to the confidence in its activity. Our approach does not require preliminary selection of well-performing pharmacophore models. Even simple pharmacophores that match many inactive compounds can be considered within the set of models used for screening. Their influence on obtained results is negligible. As a disadvantage of the proposed approach, we should mention its dependency on a calibration set and possible applicability domain issues, as transferability of calibration set probabilities to a test set may be poor. However, validation of pharmacophore models on known compounds is required almost for all pharmacophore screening approaches to select the most reliable models.

### 2.2. Benchmarking Studies

We compared the proposed approach with the following:

- the common hit approach, which ranks compounds according to the number of matched pharmacophore models;
- the commonly used OR-consensus strategy, which uses a set of pharmacophore models demonstrated reasonable performance on a dataset known of active and inactive compounds and selects compounds matching at least one of these models. OR-consensus selects compounds that are predicted as active, but cannot rank them.

We developed pharmacophore models using *psearch* software [13] and datasets of compounds retrieved from the ChEMBL database. For each protein target, we obtained from 4 to 270 pharmacophores. Precision of individual models was estimated on compounds that were not included in a training set of a corresponding model. Afterwards, we performed virtual screening of DUD-E datasets used as external test sets in this study and calculated enrichment factors for 0.25%, 0.5%, 1%, 2%, 5%, and 10% of test set compounds retrieved by models of a particular target. Additionally, we considered not all models, but only those that have at least four features with distinct coordinates. As shown previously, the application of more complex models improves the virtual screening performance [9,10]. We also considered a baseline enrichment if all compounds retrieved by at least one of pharmacophores would be considered, so called EF_100%_ (enrichment factor). The latter value is the same for Mean, Max, and CHA. These approaches were compared with the OR-consensus, in which case only pharmacophores with precision larger than 0.5 and 0.9 were considered.

**Figure 1:**
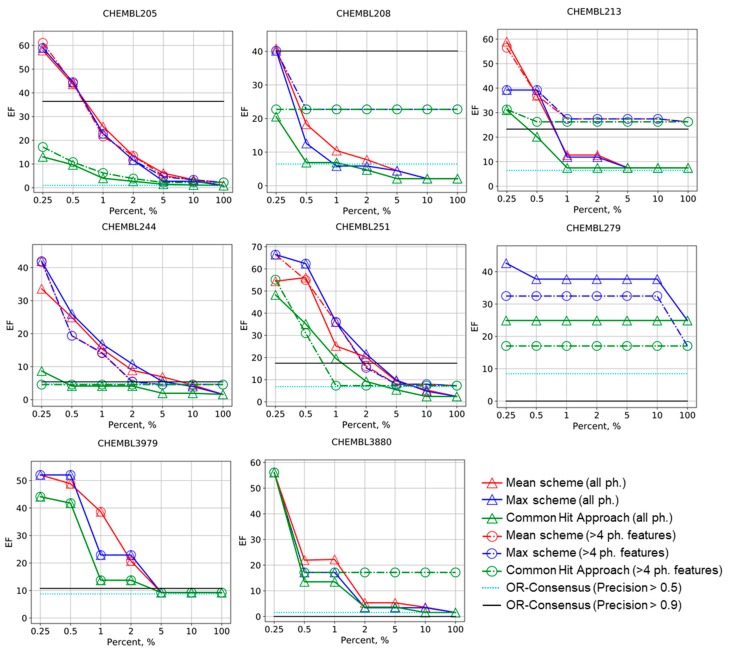
Enrichment factor (EF) curves for the Max, Mean, and common hits approach (CHA) schemes of molecules ranking in virtual screening for selected targets. Levels for OR-consensus models were given as horizontal lines (pharmacophores with precision greater than 0.5 and 0.9 are left). The numbers of corresponding ChEMBL targets are provided.

**Figure 2:**
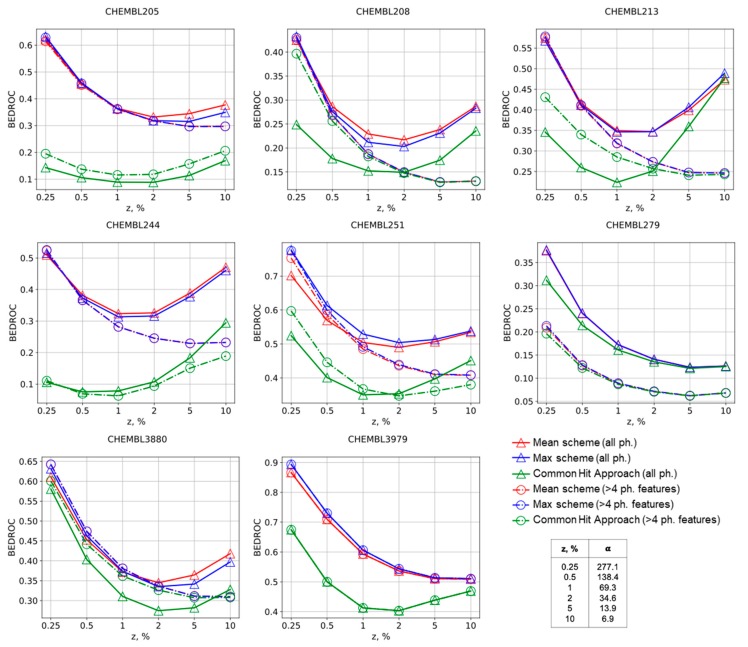
BEDROC curves for the Max, Mean, and CHA schemes of molecules ranking in virtual screening for selected targets. The numbers of corresponding ChEMBL targets are provided.

The results of the benchmarking are summarized in Figure 1 and Figure 2. Early enrichment for Mean and Max schemes is always greater than that of the OR-consensus approach if all pharmacophores are considered (OR-consensus corresponds to the level EF_100%_ in this case). The same is true if only pharmacophores with four and more features are considered; Mean and Max scheme’s early enrichment is always greater than EF_100%_. This means that ranking based on precisions of pharmacophore models on the calibrating set can be transferred to new datasets and pharmacophore models showing greater precision in selecting active compounds from the external test set more often than low-precision models. Generally, the unsupervised CHA strategy also has greater early enrichment than EF_100%_, but not always. If only high-precision pharmacophore models are considered for OR-consensus (its level is shown by light-blue and black lines), the enrichment becomes higher, but not always. In some cases, such as CHEMBL279 and CHEMBL3880 in Figure 1, no model was left, and thus enrichment dropped down to 0, whereas for CHEMBL244 and CHEMBL3979, the enrichment is not drastically different from the baseline shown by the EF_100%_ level. Thus, OR-consensus with pharmacophore selection is quite unstable. However, even if selection of high precision pharmacophores helps to increase enrichment by the OR-consensus approach, the Max and Mean schemes perform not worse or even substantially better at a small percentage of selected compounds.

One can notice that, in almost all cases, the Mean and Max schemes have better or comparable enrichment factors than CHA on the corresponding sets of pharmacophores. Our approach performs especially well in early enrichment, where it reaches very high values. This shows that the proposed Max and Mean ranking scheme based on preliminary evaluation of pharmacophore models on calibration set is better than the unsupervised CHA approach. In some examples that have not been shown in Figure 1, all approaches were failed or showed the same constant performance (see Supplementary Materials). The Max and Mean schemes perform quite similarly, but the Mean approach has slightly greater enrichment factors in some cases. The boost in performance for the Mean scheme shows that, for some cases, it is important not only to consider the most accurate pharmacophore, but also the other ones.

The selection of pharmacophores based on their complexity (only pharmacophores with more than four features were considered) had an unstable impact on the performance of both our ranking scheme and the CHA approach. For CHEMBL208, CHEMBL213, CHEMBL242, CHEMBL1871, CHEMBL3105, and CHEMBL3880, it improves the enrichment factors, sometime substantially. In CHEMBL279, CHEMBL235, and CHEMBL2971, the selection of pharmacophores led to lowering of performance for all approaches. For CHEMBL2971, the enrichment factor reached 0 at all percentages; for this dataset, only five pharmacophore models were generated and all of them were quite simple. For CHEMBL205, CHEMBL206, CHEMBL244, CHEMBL235, CHEMBL251, and CHEMBL3979, no obvious effect of pharmacophore selection was observed.

The same conclusions could be made comparing other virtual screening performance metrics. The Mean and Max approaches always have greater values of BEDROC metric in comparison with the CHA approach, thus supporting that our approach is better suitable for virtual screening, Figure 2. Selection of pharmacophore models according to number of features usually has a negative influence on BEDROC for the Mean and Max approaches, but slightly improves virtual screening by the CHA approach for early enrichment. AUC ROC, BEDROC, precision, and recall curves given in the Supplementary Materials for all studied targets generally support these findings as well.

## 3. Materials and Methods

For the development of ligand-based pharmacophore models, we collected data from the ChEMBL database (version 23) [14]. We selected targets for which we could collect reasonably large datasets of compounds with measured binding affinity expressed as K_i_, K_d_, or IC_50_ values in mol/l. Structures of retrieved compounds were curated using Chemaxon Standardizer and StructureChecker utilities [15]. Salts and other small components were removed, compounds were neutralized and tautomerized, and isotopes were removed. Additionally, we removed structures that did not pass RDKit [16] sanitization checks. After that, duplicated structures were identified. The curation workflow is available at the following link: https://bitbucket.imtm.cz/projects/STD/repos/std. We transformed affinity values to the logarithmic scale. Compounds were attributed to the active class if their affinity was ≥6 log units and to the inactive class otherwise. Duplicated structures that were attributed to active and inactive classes were removed. The list of targets and the number of retrieved compounds for final datasets are given in Table 1.

### 3.1. DUD-E Data Sets

For validation of developed models, we collected data from DUD-E datasets [17]. These data sets contained confirmed active compounds taken from ChEMBL and decoys, compounds that were not tested against a particular target and were selected from commercially available compounds by similarity of their physicochemical properties to selected active compounds. Datasets may contain different tautomeric forms of compounds and were treated as separate compounds during virtual screening. We identified and removed compounds from DUD-E datasets that were identical to compounds in collected ChEMBL datasets to avoid overestimation of model performance. The number of actives and decoys is given in Table 1.

### 3.2. Pharmacophore Modeling and Virtual Screening

We created ligand-based pharmacophore using the previously developed *psearch* approach [13]. Within this approach, we generated all possible stereoisomers for compounds with undefined stereocenters and up to 100 conformers for each compound or stereoisomer. Enumerated stereoisomers were considered as a single compound during the modeling and validation stages. Compounds were clustered according to their 2D pharmacophore representation, expecting that compounds with similar binding modes would be grouped in the same clusters. Five active and five inactive compounds were taken as a training set from each individual cluster to create pharmacophore models. Additionally, centroids of clusters were used to create a training set to capture the overall binding mode of compounds from a data set. Therefore, multiple training sets were created for each target dataset. Pharmacophore models of maximum complexity, having a maximum number of features, were generated for each training set using *psearch* software [13]. Multiple pharmacophore models could be generated for particular training sets and all of them created a set of models for an individual target (Table 2). In our study, we considered two cases: virtual screening based on all models or only on models having at least four features with distinct coordinates.

Compounds that were not included in a particular training set for a given pharmacophore model were used as a calibration set to estimate model performance. We performed virtual screening of calibration sets using the same *psearch* tool and calculated the precision of individual models.

External validation was done using the DUD-E dataset. We generated up to 100 conformers for each compound of the dataset. Enumeration of stereoisomers was not required because all compounds had defined stereocenters in DUD. The created databases of conformers of DUD-E compound were screened against developed pharmacophore models using *psearch*.

AUC ROC and BEDROC [18] were calculated using corresponding functions of RDKit [16]. When these values were calculated, all compounds were considered; those that were not selected by pharmacophore models were assigned a probability equal to zero. For enrichment, precision and recall calculation only compounds that are selected by at least one pharmacophore model are considered. For BEDROC calculation, alpha values were calculated using Equation (47) from the original publication [18]. BEDROC values for selection of 100% compounds cannot be calculated as the alpha value should be set to zero.

## 4. Conclusions

In this work, we show that pharmacophores can be treated as a special case of one-class classification machine learning models. The confidence of bioactivity prediction can be assessed on the basis of calibration set of compounds with known bioactivity. Two approaches (Max and Mean) were proposed for assigning probability value that a molecule will possess a given activity class. These approaches to compound ranking based on their matching of multiple pharmacophore models demonstrated high performance in early enrichment and works comparable or better than the reference approaches, common hits approach, and consensus of pharmacophore models based on OR boolean operator (OR-consensus). Our suggested approaches are not very sensitive to poor performing pharmacophores in a set of models compared with the OR-consensus approach and can be easily implemented within pharmacophore-based virtual screening workflows.

## Figures and Tables

**Table 1:** ChEMBL datasets used for ligand-based pharmacophore modeling and calibration and DUD-E dataset used for external validation.

ChEMBL ID	Target Name	Number of Compounds (ChEMBL)			Number of Compounds (DUD-E)		
		Actives	Inactives	Total	Actives	Inactives	Total
CHEMBL205	Carbonic anhydrase II	1394	2382	3776	492	31,172	31,664
CHEMBL206	Estrogen receptor alpha	395	1442	1837	383	20,685	21,068
CHEMBL208	Progesterone receptor	448	848	1296	293	15,650	15,943
CHEMBL213	Beta-1 adrenergic receptor	155	482	637	247	15,850	16,097
CHEMBL235	Peroxisome proliferator-activated receptor gamma	228	1052	1280	484	25,300	25,784
CHEMBL239	Peroxisome proliferator-activated receptor alpha	121	788	909	373	19,399	19,772
CHEMBL242	Estrogen receptor beta	477	972	1449	367	20,199	20,566
CHEMBL244	Coagulation factor X	676	2009	2685	537	28,325	28,862
CHEMBL251	Adenosine 2a receptor	1476	2276	3752	482	31,550	32,032
CHEMBL279	Vascular endothelial growth factor receptor 2	139	4627	4766	409	24,950	25,359
CHEMBL284	Dipeptidyl peptidase IV	281	2277	2558	533	40,950	41,483
CHEMBL1862	Tyrosine-protein kinase ABL	411	1515	1926	182	10,750	10,932
CHEMBL1871	Androgen Receptor	586	967	1553	269	14,350	14,619
CHEMBL1994	Mineralocorticoid receptor	102	532	634	94	5150	5244
CHEMBL2971	Tyrosine-protein kinase JAK2	131	2545	2676	107	6500	6607
CHEMBL3105	Poly [ADP-ribose] polymerase-1	259	1138	1397	508	30,050	30,558

**Table 2:** The number of models generated for individual targets.

ChEMBL ID	Target Name	Number of Models	Number of Models with Number of Features ≥ 4 ^a^
CHEMBL205	Carbonic anhydrase II	270	260
CHEMBL206	Estrogen receptor alpha	27	26
CHEMBL208	Progesterone receptor	37	32
CHEMBL213	Beta-1 adrenergic receptor	19	17
CHEMBL235	Peroxisome proliferator-activated receptor gamma	31	26
CHEMBL239	Peroxisome proliferator-activated receptor alpha	15	15
CHEMBL242	Estrogen receptor beta	61	53
CHEMBL244	Coagulation factor X	45	35
CHEMBL251	Adenosine A2a receptor	110	101
CHEMBL279	Vascular endothelial growth factor receptor 2	12	11
CHEMBL284	Dipeptidyl peptidase IV	34	34
CHEMBL1862	Tyrosine-protein kinase ABL	27	27
CHEMBL1871	Androgen Receptor	50	48
CHEMBL1994	Mineralocorticoid receptor	6	6
CHEMBL2971	Tyrosine-protein kinase JAK2	4	1
CHEMBL3105	Poly [ADP-ribose] polymerase-1	43	40

^a^ Number of features having distinct coordinates.

## Supplementary Materials

The following are available online, Figure S1: Enrichment curves for Max, Mean, and CHA scheme for molecules ranking in virtual screening; Figure S2: Precision curves for Max, Mean, and CHA scheme of molecules ranking in virtual screening for selected targets; Figure S3: Recall curves for Max, Mean, and CHA schemes of molecules ranking in virtual screening for selected targets; Figure S4: Histogram of BEDROC values for Max, Mean, and CHA schemes of molecules ranking in virtual screening for selected targets; Figure S5: Histogram of areas under ROC curve (AUC) values for Max, Mean, and CHA schemes of molecules ranking in virtual screening for selected targets; Figure S6: ROC curves for Max, Mean, and CHA schemes of molecules ranking in virtual screening for selected targets.
