# Supplementary material for: Probabilistic Approach for Virtual Screening Based on Multiple Pharmacophores
Source: Molecules. 2020 Jan 17;25(2):385. doi: 10.3390/molecules25020385 (PMC7024325; doi:10.3390/molecules25020385)
Supplement: Supplementary file 1 [file molecules-25-00385-s001.zip › molecules-675707-SI.pdf]

Probabilistic Approach for Virtual Screening Based on Multiple Pharmacophores

Timur I. Madzhidov [1], Assima Rakhimbekova [1], Alina Kutlushuna [1,2], and Pavel Polishchuk [2,*]

[1] A.M. Butlerov Institute of Chemistry, Kazan Federal University, Kremlevskaya Str. 18, 420008 Kazan, Russia; timur.madzhidov@kpfu.ru (T.M.)

[2] Institute of Molecular and Translational Medicine, Faculty of Medicine and Dentistry, Palacky University and University Hospital in Olomouc, Hnevotinska 5, 77900 Olomouc, Czech Republic; pavlo.polishchuk@upol.cz (P.P.), alina.kutlushina@upol.cz (A.K.), olena.mokshyna@upol.cz (O.M.)

* Correspondence: pavlo.polishchuk@upol.cz; Tel.: +420-585632298 (P.P.)

Table of content:

Figure S1: Enrichment curves for Max, Mean and CHA scheme for molecules ranking in virtual screening.
Figure S2: Precision curves for Max, Mean, and CHA scheme of molecules ranking in virtual screening for selected targets.
Figure S3: Recall curves for Max, Mean, and CHA scheme of molecules ranking in virtual screening for selected targets.
Figure S4: Histogram of BEDROC values for Max, Mean, and CHA scheme of molecules ranking in virtual screening for selected targets.
Figure S5: Histogram of areas under ROC curve (AUC) values for Max, Mean, and CHA scheme of molecules ranking in virtual screening for selected targets.
Figure S6: ROC curves for Max, Mean, and CHA scheme of molecules ranking in virtual screening for selected targets.
Table S1: Values of performance metrics for all approaches.

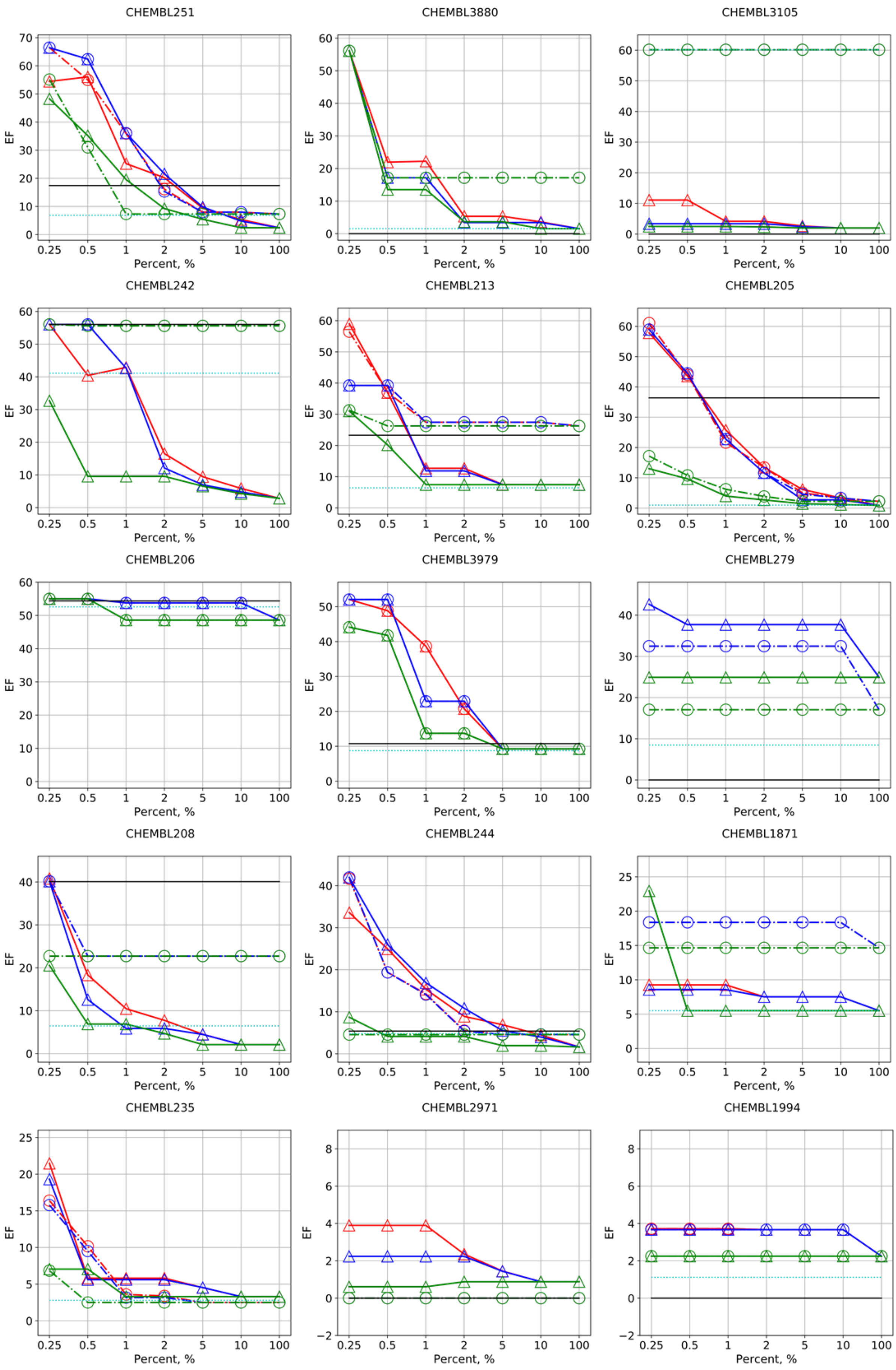

CHEMBL251
CHEMBL3880
CHEMBL3105
CHEMBL242
CHEMBL213
CHEMBL205
CHEMBL206
CHEMBL3979
CHEMBL279
CHEMBL208
CHEMBL244
CHEMBL1871
CHEMBL235
CHEMBL2971
CHEMBL1994
EF
Percent, %
0.25
0.5
1
2
5
10
100

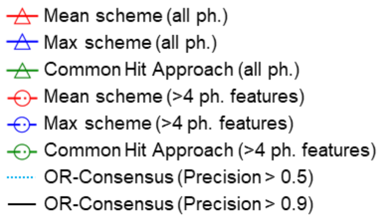

Figure S1. Enrichment curves for Max, Mean scheme and Common Hit Approach for molecules ranking in virtual screening. Levels for OR-consensus models are given (pharmacophores with precision greater than 0.5 and 0.9 are left). The number of corresponding ChEMBL bioassay is provided. Compounds that were not selected by any pharmacophore models were ignored and not considered in enrichment computation.

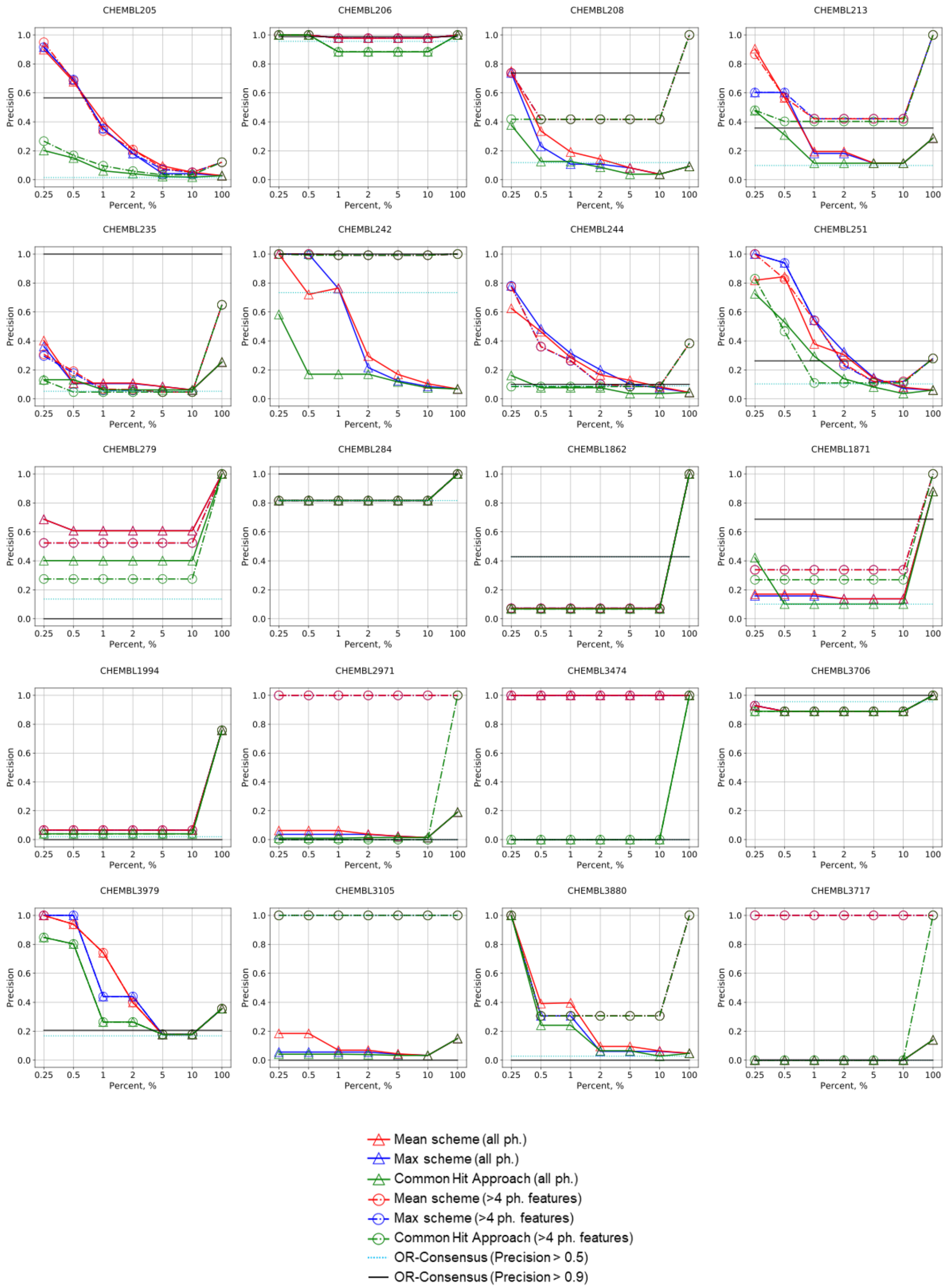

Figure S2. Precision curves for Max, Mean, and CHA scheme of molecules ranking in virtual screening for selected targets. OR-consensus (pharmacophores with precision greater than 0.5 and 0.9 are left) values are given for comparison. Percent of selected compounds is given as argument. The numbers of corresponding ChEMBL targets are provided. Compounds that were not selected

by any pharmacophore models were ignored and not considered in precision computation. Thus, precision at 100% (all compounds selected by at least one pharmacophore models are considered as predicted active) is not equal to 1.

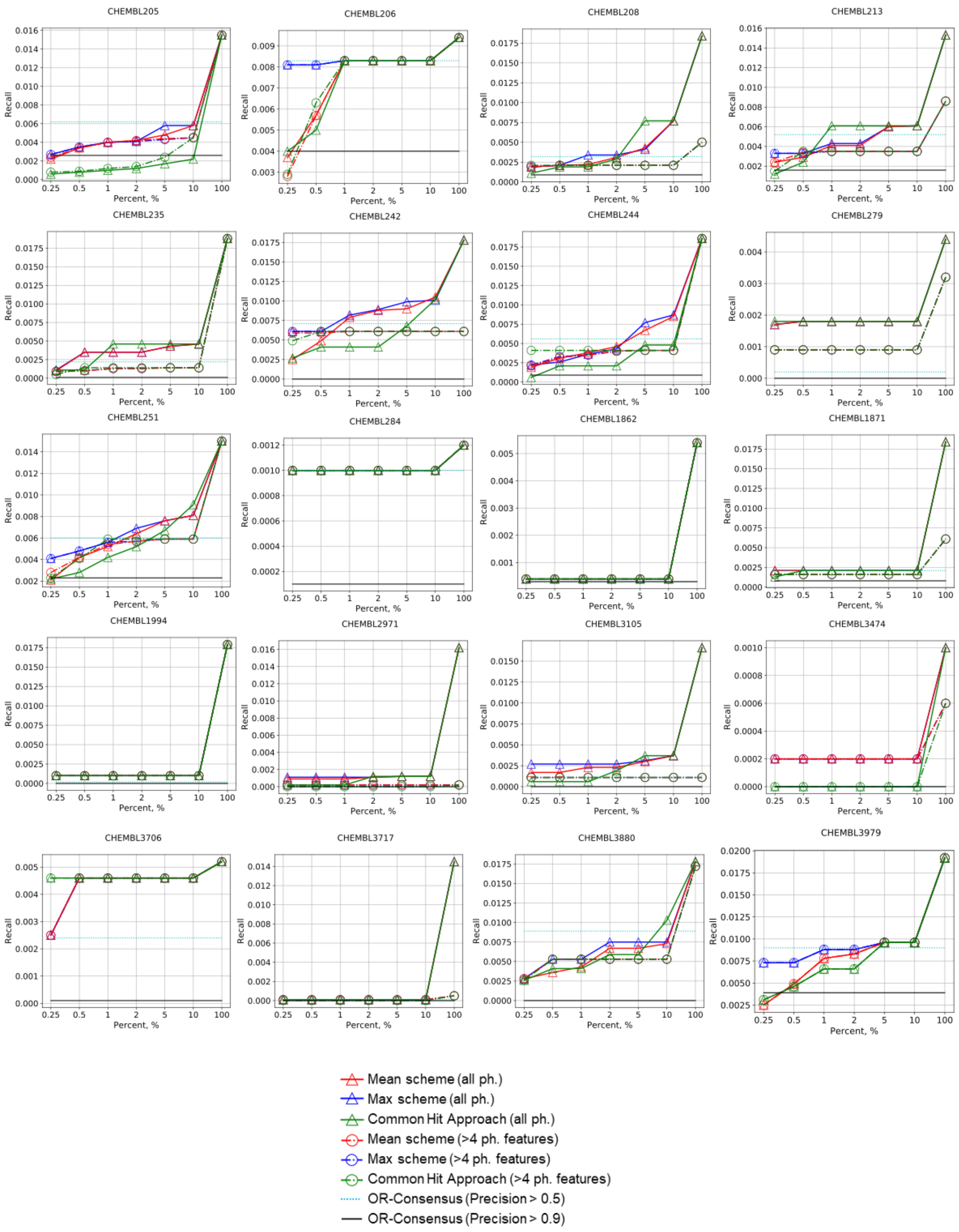

Figure S3. Recall curves for Max, Mean, and CHA scheme of molecules ranking in virtual screening for selected targets. OR-consensus (pharmacophores with precision greater than 0.5 and 0.9 are left) values are given for comparison. Percent of selected compounds is given as argument. The numbers of corresponding ChEMBL targets are provided. Compounds that were not selected by any pharmacophore models were ignored and not considered in recall computation.

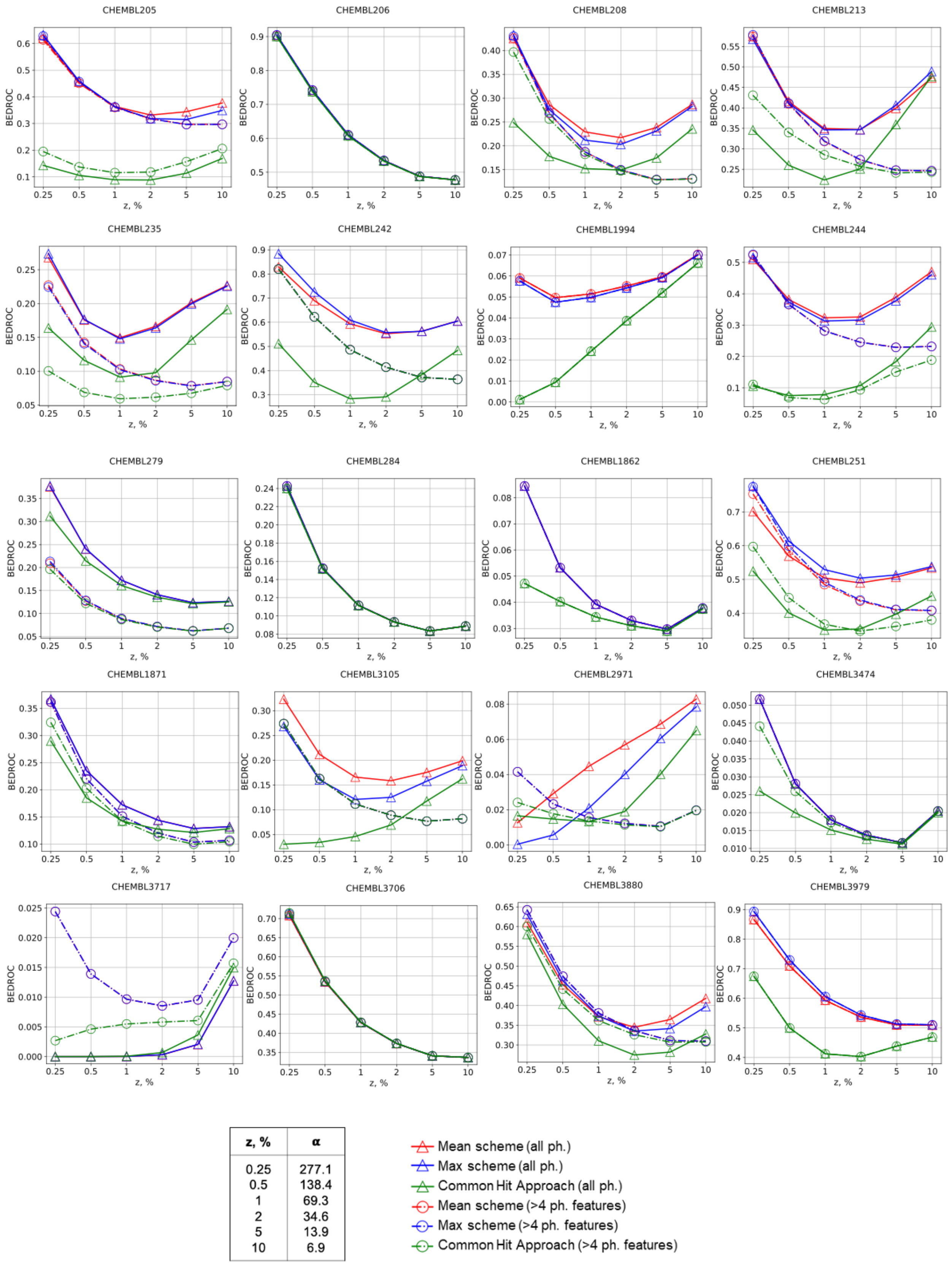

z, %	α
0.25	277.1
0.5	138.4
1	69.3
2	34.6
5	13.9
10	6.9

Figure S4. BEDROC curves for Max, Mean, and CHA scheme of molecules ranking in virtual screening for selected targets. Alpha values for BEDROC computation were adjusted for every ratio of selected compounds (z) based on equation 47 from [Truchon J.-F., Bayly C.I. Evaluating Virtual Screening Methods: Good and Bad Metrics for the "Early Recognition" Problem//J. Chem. Inf. Model. 2007. Vol. 47, № 2. P. 488–508]. Compounds that were not selected by any pharmacophore

models are considered as having probability of activity equal to zero. The numbers of corresponding ChEMBL targets are provided.

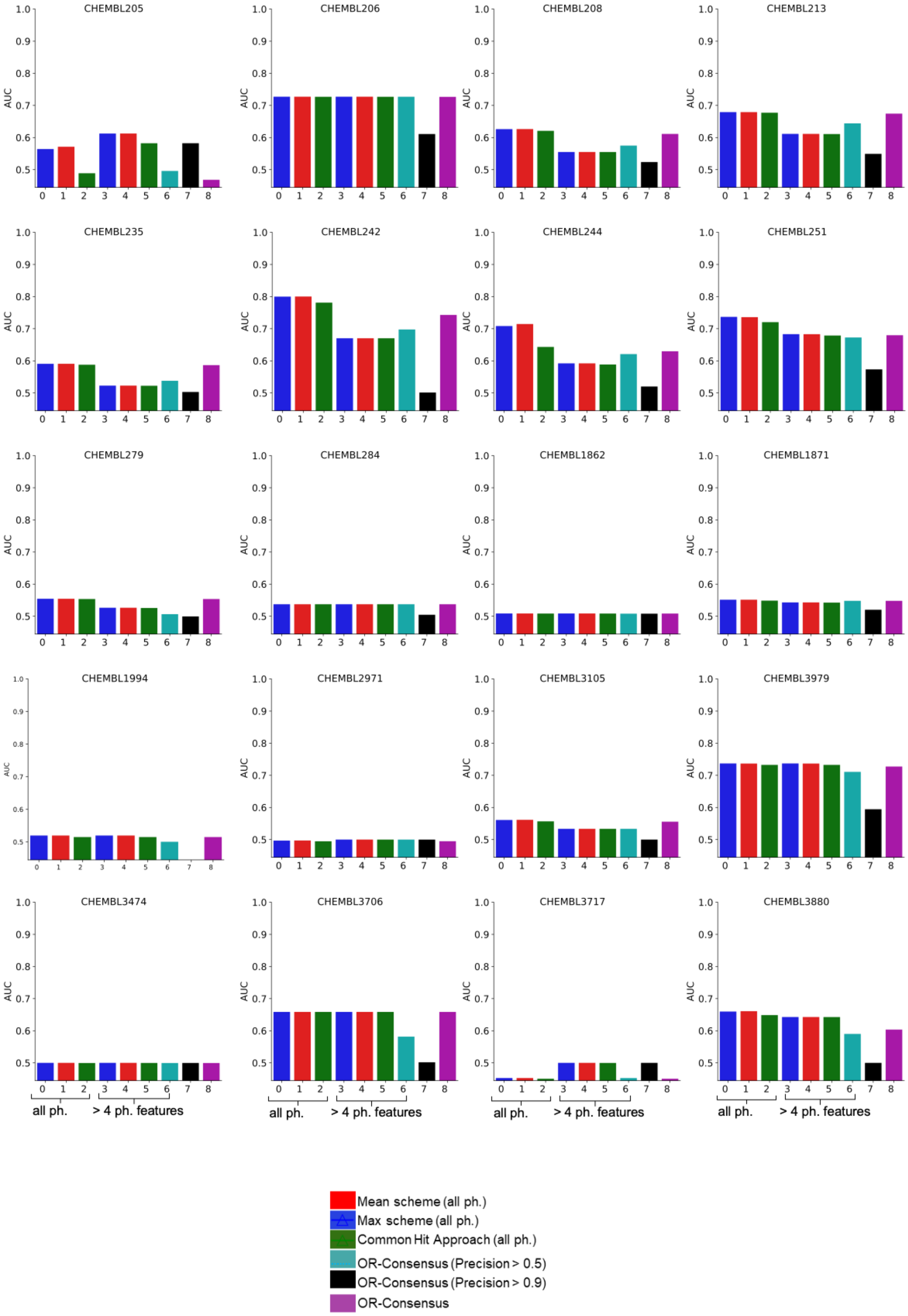

Figure S5. Histogram of areas under ROC curve (AUC) values for Max, Mean, and CHA scheme of molecules ranking in virtual screening for selected targets. OR-consensus (pharmacophores with

precision greater than 0.5 and 0.9 are left) values are given for comparison. Notice that in CHEMBL1994 no pharmacophores with precision greater than 0.9 were left and corresponding value was excluded. Compounds that were not selected by any pharmacophore models are considered as having probability of activity equal to zero. The numbers of corresponding ChEMBL targets are provided.

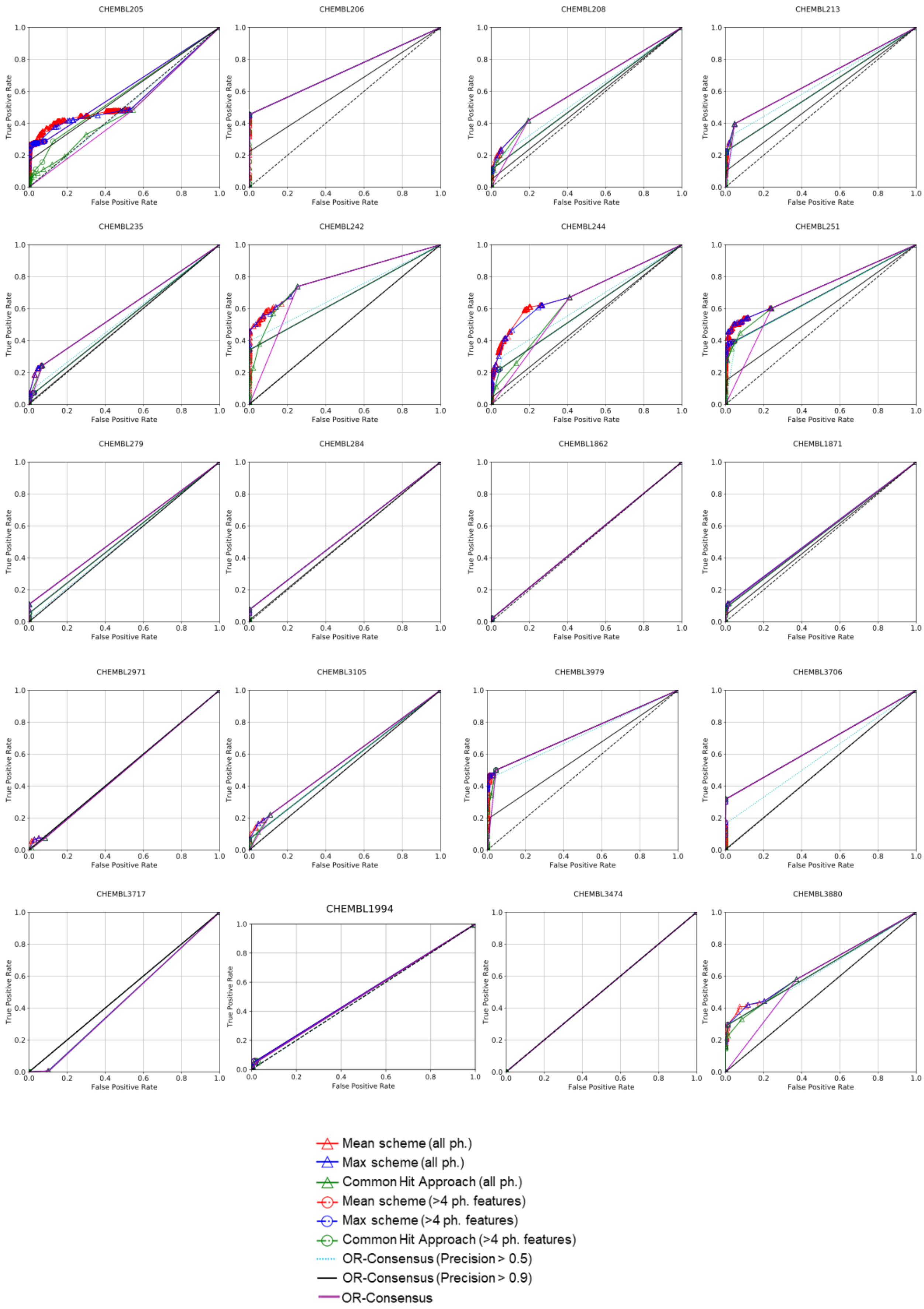

Figure S6. ROC curves for Max, Mean, and CHA scheme of molecules ranking in virtual screening for selected targets. OR-consensus (pharmacophores with precision greater than 0.5 and 0.9 are left) values are given for comparison. Notice that in CHEMBL1994 no pharmacophores with precision

greater than 0.9 were left and corresponding value was excluded. Compounds that were not selected by any pharmacophore models are considered as having probability of activity equal to zero. The numbers of corresponding ChEMBL targets are provided.
